# The Impact of Allergic Rhinitis on Australian Adults in an Urban Setting: A Qualitative Exploration

**DOI:** 10.3390/jcm15187284

**Published:** 2026-09-19

**Authors:** Ayomikun Opadokun, Ereeny Mikhail, Luke X. Chong, Cenk Suphioğlu, Moneisha Gokhale, Serap Azizoğlu

**Affiliations:** 1 School of Medicine (Optometry), Faculty of Health, Deakin University, Waurn Ponds, Geelong, VIC 3216, Australia; s222248537@deakin.edu.au (A.O.); e.mikhail@deakin.edu.au (E.M.); luke.chong@deakin.edu.au (L.X.C.); moneisha.gokhale@deakin.edu.au (M.G.); 2 NeuroAllergy Research Laboratory (NARL), School of Life and Environmental Sciences, Deakin University, Waurn Ponds, Geelong, VIC 3216, Australia; cenk.suphioglu@deakin.edu.au; 3 Institute for Mental and Physical Health and Clinical Translation (IMPACT), Deakin University, Geelong, VIC 3216, Australia; 4 Centre for Sustainable Bioproducts (CSB), Deakin University, Geelong, VIC 3216, Australia

**Keywords:** allergic rhinitis, hay fever, allergy, quality of life, burden

## Abstract

**Background/Objectives:** Allergic rhinitis (AR) is a prevalent and chronic condition that is frequently underestimated in clinical practice, despite its substantial impact on quality of life (QoL). While epidemiological studies have quantified the prevalence, risk factors and symptom burden, limited research has explored the lived experiences of adults living with AR in Australia, and more specifically in Victoria. This study aimed to explore the lived experiences of adults with AR in urban Victoria, Australia. **Methods:** A qualitative study utilising online semi-structured in-depth interviews was conducted with thirty-eight adults residing in Melbourne and Geelong, Victoria, Australia. Data were analysed using reflexive thematic analysis as described by Braun and Clarke. **Results:** Of the 38 participants, 57.9% (*n* = 22) were self-diagnosed and 42.1% (*n* = 16) had received a professional diagnosis of AR. The following five themes were constructed: 1. AR is shaped by onset, family history and comorbidities; 2. Environmental exposures and seasonal changes strongly influence the experience of AR patients; 3. Coping with AR involves a mix of lifestyle adjustments, medication and personal adaptive strategies; 4. AR disrupts QoL through poor sleep, emotional strain, social limitations and reduced productivity; and 5. Limited awareness and barriers in healthcare access hinder effective management of AR. **Conclusions:** Participants described AR as extending beyond physical symptoms to emotional, social and economic impacts shaped by multiple factors. These complex lived experiences emphasise the need for more tailored management and improved clinical recognition of the psychosocial burden of AR.

## 1. Introduction

Allergic rhinitis (AR), commonly known as hay fever, is an IgE-mediated inflammatory disorder of the nasal mucosa (i.e., upper airways), triggered by otherwise harmless environmental allergens such as pollen, dust mites and animal dander [1,2]. It is one of the most prevalent chronic respiratory conditions worldwide, affecting an estimated 10–30% of the global population [3,4]. In Australia, the prevalence of AR has nearly doubled between 2001 and 2022, with almost one in four adults (23.9%) affected nationally, and the rates are the second highest in the state of Victoria at 29.8% [5]. 

Rapid urbanisation, pollution and climate variability have contributed to rising prevalence rates [6,7,8]. Specifically, in Australia, studies have linked climate-related increases in pollen exposure and air pollution to higher AR prevalence [9]. Urban living increases exposure to airborne pollutants while reducing contact with environmental biodiversity, leading to altered immune tolerance and heightened allergic sensitisation [10,11]. Additionally, vehicle and industrial emissions can chemically modify pollen, increasing its allergenicity and capacity for deep airway penetration [12,13,14]. These environmental interactions exacerbate both the incidence and severity of AR symptoms.

AR commonly coexists with asthma and may contribute to substantial respiratory morbidity, while its chronic and relapsing nature alone can substantially impair quality of life (QoL) on a regular basis [15]. Sleep disturbance, fatigue, reduced concentration and mood changes are common [16,17], while stigma and social withdrawal can further diminish wellbeing [18,19]. In children and adolescents, AR has been associated with poorer academic performance and disrupted sleep [20,21], which can negatively affect cognitive functioning and school attendance. The economic consequences of AR are equally significant. Absenteeism and lack of productivity at work or school, when present, lead to reduced efficiency and increased indirect costs [22,23]. The cost of allergy medications and over-the-counter treatments in Australia represents a notable out-of-pocket burden for many patients [24]. Despite this multidimensional burden, AR remains under-recognised and undertreated in primary care [24], and patients frequently rely on self-directed management [25].

Research on AR has traditionally focused on the use of quantitative symptom scales and QoL indices, such as the Rhinoconjunctivitis Quality of Life Questionnaires (RQLQ) [26]. While these tools enable standardised measurement of symptoms and QoL, they offer only partial insight into the complex emotional and social realities of living with AR. By contrast, qualitative studies in other chronic conditions have elucidated nuanced coping strategies and identity-related challenges that have been overlooked in quantitative methods [27]. However, within the Australian context, qualitative investigations of allergic disease, and specifically AR, remain scarce [15,19,28,29]. To address this gap, the present study explores the lived experiences of adults with AR in urban Victoria, Australia, which has been anecdotally named the allergy capital of the world. By foregrounding patients’ perspectives within their environmental and social contexts, this research seeks to inform more patient-centred approaches to AR management and urban public health policy.

## 2. Materials and Methods

### 2.1. Study Design

This qualitative study used in-depth semi-structured interviews to explore the lived experiences of adults with AR living in urban Victoria, Australia. Ethics approval was obtained from the Human Ethics Advisory Group—Health, Deakin University (HEAG ID: 2024/HE000829). Interviews were conducted individually via Zoom (Zoom Video Communications, Inc., San Jose, CA, USA) and audio recorded; recordings were stored on encrypted drives and deleted after transcription. The study was conducted over June to October in 2025, which falls into the winter to spring period in southern Australia.

### 2.2. Participants and Sampling

Purposive sampling was used to ensure participants had genuine, first-hand lived experience of AR and were able to provide rich, in-depth accounts of that experience [30], rather than being targeted at any demographic subgroup. Participants were recruited, as per the human ethics approval, through flyers, social media and word-of-mouth, with no single channel prioritised. Snowball sampling was additionally employed to broaden recruitment scope beyond the initial pool of participants and to reach individuals who may not have been accessible through flyer or social media-based recruitment alone [31]. This combination of recruitment methods was deliberately designed to reduce reliance on any single channel and to capture a more diverse range of participants across urban Victoria.

Inclusion criteria were: (1) age 18 years or older, (2) self-reported or professionally diagnosed seasonal AR, and (3) residence in urban Melbourne or Geelong, Victoria, Australia (population > 250,000). Individuals whose primary health concern was not AR (i.e., whose interview account was substantively dominated by another health condition rather than by AR) were excluded.

Consistent with reflexive thematic analysis, the sample size was guided by the concept of data adequacy where the dataset is rich and varied enough to support meaningful theme development for the research question, rather than a fixed numerical target [32,33]. The final sample provided rich and varied accounts of symptom experience, management, and social impact, which was judged sufficient to support meaningful theme development for the aims of this study.

### 2.3. Interview

Interview design was theoretically informed by the revised Wilson and Cleary model of health-related QoL (HRQoL) proposed by Ferrans et al. [34], which conceptualises QoL as a dynamic process linking biological function, symptom experience, functional status, general health perceptions and overall QoL, shaped by individual and environmental characteristics. This framework guided development of the interview guide (Supplementary Material S1) to move beyond symptom description and capture the complex relationship of an individual’s lived experience with AR. Questions explored demographics and background, symptom experiences and emotional and social consequences, impacts on work and daily activities, coping strategies and perceptions of environmental and healthcare influences, consistent with the framework’s core domains. Open-ended questions and prompts were used to encourage reflection and elaboration, allowing participants to foreground aspects of AR most salient to their lived experience, while maintaining conceptual consistency across interviews. Two pilot interviews were conducted to refine wording and sequencing; as only minor refinements were required, these interviews were retained in the final analysis. 

All interviews were conducted by the first author (AO), who has a background in biomedical science. As part of her Honours training, AO successfully completed a Research Methods subject, which included a qualitative research component. AO also received guidance in semi-structured interviewing from EM, who has experience and training in qualitative health research. AO had a pre-existing relationship with a subset of participants prior to the study while the remaining participants had no prior relationship with the interviewer. Written informed consent was obtained from all participants prior to the interview. Each participant was interviewed once with only the researcher and participant present, and no repeat interviews were conducted. Field notes were taken during and after interviews to record contextual observations. Interviews were audio-recorded and transcribed verbatim manually by AO. No transcripts or findings were returned to participants.

### 2.4. Data Analysis

Qualitative data were analysed using reflexive thematic analysis (RTA), following Braun and Clarke’s six phases: (1) familiarisation with the data, (2) coding, (3) generating initial themes, (4) reviewing themes, (5) defining and naming themes and (6) reporting. Reflexive awareness of researcher positionality was maintained throughout data collection and analysis. The first author (AO) read each transcript multiple times and recorded early analytic impressions in a reflexive journal. Manual coding identified both semantic (explicit) and latent (conceptual) meanings. A second researcher (EM), who has qualitative health research experience and training, independently coded a subset of transcripts. The two researchers then compared and discussed their coding. No formal coder-agreement or consensus procedure was used. Where interpretations differed, these were discussed as alternative readings of the data and used as an analytical resource to inform theme development. Initial themes were generated and further refined by the authors. Themes were reviewed for internal coherence and distinctiveness and were defined to reflect conceptual clarity and analytic depth [35].

RTA was chosen for its flexibility, emphasis on reflexivity and suitability for health experience research [36]. Our analytic approach broadly aligns with a constructionist orientation, which is compatible with reflexive thematic analysis, in which themes are understood as shaped through the researchers’ engagement with the data rather than as objective categories waiting to be discovered [35]. A hybrid approach was adopted overall: the interview guide was deductively informed by the Wilson and Cleary model, shaping the broad domains explored with participants, while coding and theme development were conducted inductively from participants’ own accounts. As the interview guide was structured around the Wilson and Cleary model’s domains, the resulting themes reflect some conceptual overlap with this framework; this is a feature of the study’s design rather than a deductive application of the framework during coding.

Rigour was supported through attention to Lincoln and Guba’s [37] trustworthiness criteria: credibility, dependability, confirmability and transferability. An audit trail documented all analytic decisions, including coding notes, memos and theme maps. Research team debriefing and reflexive journaling supported interpretive transparency and critical reflection on how researcher positioning shaped the analysis [38]. Reflexive memos addressed how the researcher’s disciplinary background could influence interpretation [35]. Reporting followed the Consolidated Criteria for Reporting Qualitative Research (COREQ) checklist (Supplementary Material S2) [39] to support transparent and comprehensive reporting. The checklist covers three domains: (1) research team and reflexivity, (2) study design and (3) data analysis and reporting. It guides the reporting of this study.

During the preparation of this work, the authors used Microsoft Copilot Version number: 2.20260916.60.0 for grammatical editing and language refinement. The authors reviewed and edited the output as needed and take full responsibility for the content of the published article.

## 3. Results

### 3.1. Participant Demographics

A total of 39 participants were interviewed. There were no refusals to participate. One participant was excluded from data analysis because their primary health concern was not AR, resulting in a final sample of 38 participants. The mean ± standard deviation age of participants was 31.4 ± 13.5 years (range 18–72 years). Interviews lasted 30 to 60 minutes and were audio-recorded with consent and transcribed verbatim. Participants were predominantly female (68.4%, *n* = 26), while 31.6% (*n* = 12) were male. Of the 38 participants, 57.9% (*n* = 22) were self-diagnosed and 42.1% (*n* = 16) had received a professional diagnosis of AR. 

### 3.2. Themes

**Figure 1:**
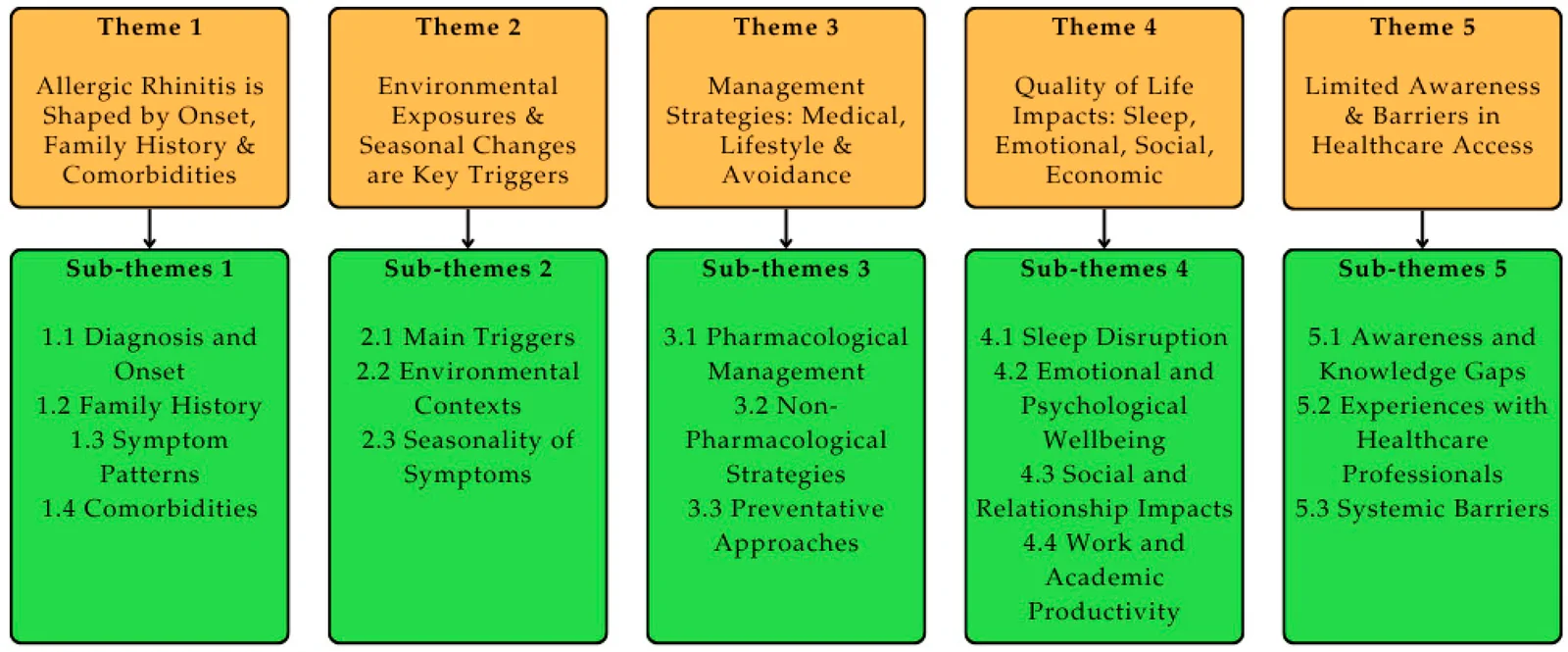
Thematic map of participants’ experiences of allergic rhinitis, illustrating the five major themes and their sub-themes.

Five themes were constructed from the data: (1) AR is shaped by onset, family history and comorbidities; (2) Environmental exposures and seasonal changes strongly influence the experience of AR patients; (3) Coping with AR involves a mix of lifestyle adjustments, medication and personal adaptive strategies; (4) AR disrupts QoL through poor sleep, emotional strain, social limitations and reduced productivity; and (5) Limited awareness and barriers in healthcare access hinder effective management of AR. Representative quotes are embedded within each theme description; additional supporting quotes are presented in Table 1, Table 2, Table 3, Table 4 and Table 5. An overview of the themes and sub-themes is presented in Figure 1.

#### 3.2.1. Theme 1: Allergic Rhinitis Is Shaped by Onset, Family History and Comorbidities

AR was described as a deeply personal and evolving condition, shaped by the timing of onset, family history and coexisting allergic or respiratory illnesses.

##### Diagnosis and Onset

Participants had varied experiences with their diagnosis of AR, with most being self-diagnosed and some having a formal clinical diagnosis. Some professionally diagnosed participants described greater awareness of specific triggers: *“I’ve had allergy testing twice—conclusive for dust mites and different hay”* (P7). Some participants described symptom onset in childhood: *“I was diagnosed when I was really little, maybe around four years old, because my nose was constantly blocked and I couldn’t sleep properly”* (P5). However, others described a more gradual process of self-diagnosis, only realising patterns of recurrence after years of misattribution.

##### Family History

Family history shaped participants’ understanding and management of AR. For some, observing relatives manage similar symptoms offered a framework for self-care: *“My mum always had tissues and eye drops in her bag, so I kind of grew up knowing what to do”* (P14). In contrast, those without a family history of AR described feeling uncertain and isolated, reinforcing the role of family as both a source of practical knowledge and emotional validation.

##### Symptom Patterns

Participants reported a wide range of symptoms including nasal congestion, sneezing, itchy and watery eyes, headaches and fatigue. The variability in intensity and timing shaped their perception of the illness: *“It’s unpredictable, some days it’s just a bit of sneezing, other days it’s full-blown congestion, watery eyes and exhaustion”* (P19). These patterns reflected AR as a fluctuating but relentless presence demanding constant vigilance.

##### Comorbidities

Participants described living with comorbid conditions, such as asthma and eczema, which amplified the burden of AR: *“I’ve got asthma as well, so when the hay fever starts, my breathing gets worse. It’s like a chain reaction”* (P6). Participants often viewed their allergic conditions as a single integrated system of vulnerabilities, reinforcing the need for holistic and coordinated care. Managing overlapping conditions required continuous effort and emotional endurance for some participants: *“It feels like I’m always treating something; nose spray, asthma puffer, creams. There’s never a break,”* said one participant (P8). Such comorbidities shaped not only physical experiences but also perceptions of health identity. 

Across these accounts, AR emerged not as a fixed diagnosis but as an identity built gradually through personal and family experience, shaped by pattern recognition over time rather than a single clinical moment. Additional illustrative quotes supporting Theme 1 are presented in Table 1.

**Table 1:** Supporting participant quotes for Theme 1: AR is shaped by onset, family history and comorbidities.

Sub-Theme	Participant Quotes
Diagnosis and Onset	*“Self-diagnosed; I didn’t really identify it until I was about 18. And it is all self-managed as well”* (P1) *“I thought it was just a cold at first, until I realised it came back every spring from about Year 9.”* (P16)
Family History	*“Everyone in my family has it, mum, dad, even my little sister, we all sneeze together in spring.”* (P7) *“No one in my family had it, so at first, I thought maybe it was something else.”* (P9)
Symptom Patterns	*“…when spring comes around, I get really runny nose, itchy, watery eyes.”* (P2) *“Watery eyes, massive fatigue, blocked and runny nose, sneezing—every symptom at once sometimes.”* (P5)
Comorbidities	*“When my allergies get bad, my skin flares up too—it’s all connected.”* (P12)

AR, allergic rhinitis; P, participant.

#### 3.2.2. Theme 2: Environmental Exposures and Seasonal Changes Strongly Influence the Experience of Allergic Rhinitis Patients

Participants consistently identified environmental exposures and seasonal changes as powerful determinants of symptom severity. Urban living, air quality and climatic variability shaped their experiences in ways that often felt uncontrollable and frustrating.

##### Main Triggers

Pollen emerged as a widely recognised trigger, particularly during spring: *“When the wind picks up in spring, I can feel it instantly; my nose starts running, and I can’t stop sneezing”* (P9). For others, indoor allergens, such as dust mites and pet dander were equally aggravating, with some reporting both outdoor and indoor triggers simultaneously. These accounts highlighted that AR was not merely a seasonal nuisance but a constant adaptation to multiple environmental irritants.

##### Environmental Contexts

Participants described clear links between geographical location and symptom intensity. Melbourne was frequently cited as exacerbating symptoms: *“I noticed my allergies got worse when I moved to Melbourne—it’s like something in the air here!”* (P10). Others compared urban versus rural living: *“When I visit my parents in the country, I can breathe again. But as soon as I come back to the city, everything flares up”* (P24). Participants also described local flora, air pollution and weather as factors they perceived to aggravate their symptoms of AR.

##### Seasonality of Symptoms

Seasonal variation defined the rhythm of AR for participants, with spring identified as the most challenging period: *“September hits, and it’s like a switch flips; I’m constantly sneezing, my eyes swell, I feel drained”* (P16). Participants also described symptoms extending beyond traditional allergy seasons, with some experiencing symptoms across the year.

Participants described a loss of seasonal predictability, with pollen, urban pollutants and changing weather contributing to a sense of constant environmental vigilance. Additional illustrative quotes supporting Theme 2 are presented in Table 2.

**Table 2:** Supporting participant quotes for Theme 2: Environmental exposures and seasonal changes.

Sub-Theme	Participant Quotes
Main Triggers	*“It’s not just the pollen, it’s the dust from construction and car fumes that make it worse.”* (P21) *“If I visit a friend with cats, I’m sneezing within ten minutes.”* (P28) *“Vacuuming makes me sneeze so much, I have to wear a mask.”* (P13) *“Dust, cat hair, grass pollen and fungal spores mess me up.”* (P12)
Environmental Contexts	*“When it’s humid or when there’s a storm, my sinuses block completely.”* (P33) *“When I’m near traffic or construction sites, my eyes sting immediately.”* (P36)
Seasonality of Symptoms	*“I dread spring every year. I stock up on tissues and antihistamines like clockwork.”* (P4) *“It used to be just a few weeks, but now it lingers for months. Even in winter, I’ll get random flare-ups.”* (P22) *“It used to be just spring, but now it can hit anytime—rain, wind, even changes in temperature.”* (P23)

P, participant.

#### 3.2.3. Theme 3: Coping with Allergic Rhinitis Involves a Mix of Lifestyle Adjustments, Medications and Personal Adaptive Strategies

Participants described developing a wide range of strategies to reduce the impact of AR on daily life, reflecting both adaptive self-management and barriers to healthcare access.

##### Pharmacological Management

Oral antihistamines were commonly described as a form of symptom control, with participants learning through experience which medications or combinations of medications worked best: *“I rotate between Telfast and Zyrtec; some days one works better than the other”* (P12). Cost and accessibility were recurring barriers to optimal medication use, with pharmacological management shaped as much by financial realities as clinical need.

##### Non-Pharmacological Strategies

Cleaning routines, air filtration and physical barriers were adopted to limit exposure. One participant described adapting their workspace: *“I keep an air purifier on my desk and try not to open windows on windy days”* (P11). Others used cooling or soothing techniques and behavioural adjustments, such as showering after being outdoors, reflecting participants’ efforts to maintain a sense of control over their condition through daily habits.

##### Avoidance and Adaptive Approaches

Participants described allergen avoidance as an important component of their lifestyle adjustments, including modifications to social plans and activities: *“I avoid picnics or outdoor events when it’s windy—it’s just not worth it”* (P6). While these measures were often effective, they also restricted social life and personal freedom, highlighting the ‘trade-offs’ that individuals made to maintain control over their health. 

Across these varied strategies, participants’ shared aim was an ongoing pursuit of control over a condition they could not fully predict or eliminate. Additional illustrative quotes supporting Theme 3 are presented in Table 3.

**Table 3:** Supporting participant quotes for Theme 3: Coping strategies for AR.

Sub-Theme	Participant Quotes
Pharmacological Management	*“Sometimes the tablets don’t do anything; I just have to ride it out.”* (P29) *“I’m on immunotherapy—it helps a bit, but it’s expensive and you have to be consistent.”* (P8) *“I’d use nasal sprays more if they weren’t so pricey. You end up picking what you can afford.”* (P20)
Non-Pharmacological Strategies	*“I clean and vacuum a lot—especially the windows and blinds—it helps a bit with dust and pollen.”* (P27) *“Cold compresses over my eyes are the only thing that calms them down after a flare-up.”* (P2)
Avoidance and Adaptive Approaches	*“I just try to stay away from grass. I do more water-based activities like spearfishing and surfing, feels better.”* (P4) *“If I see the pollen count is high, I won’t hang washing outside or go for a walk.”* (P14) *“We replaced all the carpets with tiles because vacuuming made my symptoms unbearable.”* (P16)

AR, allergic rhinitis; P, participant.

#### 3.2.4. Theme 4: Allergic Rhinitis Disrupts Quality of Life Through Poor Sleep, Emotional Strain, Social Limitations and Reduced Productivity

Participants consistently described AR as an exhausting and disruptive condition that eroded QoL across physical, emotional and functional domains.

##### Sleep Disruption

Sleep disturbance emerged as a pervasive consequence of AR. Participants reported difficulty in breathing at night due to nasal congestion, with disruptions carrying over into daily functioning: *“At night, my nose blocks completely, I have to sit up to breathe, and that means I barely get any rest”* (P4). Participants described AR not as an episodic irritation but as a persistent intrusion, blurring the line between physical and cognitive exhaustion.

##### Emotional and Psychological Wellbeing

Emotionally, participants framed AR as a constant source of frustration at both the condition and its interference with daily activities: *“It’s just relentless. You can’t control it, and that makes it so frustrating”* (P2). Some expressed acceptance: *“I’ve had it so long that I just get on with [it]—it’s annoying, but you can’t fight it every day”* (P24). Others described frustration and exhaustion from constant vigilance; *“It’s draining having to think about it all the time- what to clean, what to avoid, what to take”* (P35). Embarrassment and self-consciousness were also common in public and professional settings, compounding feelings of isolation. Across accounts, coping appeared less about eliminating symptoms than about maintaining psychological balance amid chronic discomfort.

##### Social and Relationship Impacts

AR limited social participation, particularly during severe flare-ups, with the unpredictable nature of symptoms making planning difficult: *“You never know when it’s going to hit, so you stop saying yes to things”* (P26). Social withdrawal was compounded by post-COVID stigma around sneezing, with participants frequently needing to explain their symptoms to others: *“I always need to explain, ‘it’s not COVID’…people give you weird looks otherwise”* (P20).

##### Work and Academic Productivity

Participants described significant challenges maintaining performance at work and university due to persistent symptoms and fatigue. Presenteeism was common: *“You’re at work, but you’re not really functioning at full capacity. You’re just pushing through it”* (P10). These experiences revealed that AR disrupted not only comfort but also occupational identity and academic performance. 

Rather than affecting isolated domains, participants described these impacts as compounding across poor sleep, emotional strain, social participation and daily performance. Additional illustrative quotes supporting Theme 4 are presented in Table 4.

**Table 4:** Supporting participant quotes for Theme 4: AR-related disruptions to quality of life.

Sub-Theme	Participant Quotes
Sleep Disruption and Fatigue	*“Even when I sleep eight hours, I wake up drained. The congestion makes it impossible to breathe properly.”* (P11) *“You wake up exhausted, and by mid-morning your head feels heavy, your focus is gone.”* (P19) *“It builds up over days; it’s not just tiredness, it’s mental fog.”* (P25)
Emotional Impact	*“It makes me crazy…I do my makeup and it gets ruined, very frustrating.”* (P12) *“It wears you down; you start to feel helpless because nothing seems to stop it.”* (P9) *“My eyes get so red people think I’ve been crying or that I’m sick.”* (P14) *“It’s awkward sneezing all the time in meetings—people assume you’re contagious.”* (P33)
Social Impact	*“I once had to leave a cousin’s birthday party because of cats.”* (P12) *“Friends didn’t take it seriously when I cancelled plans, thought I was joking.”* (P6) *“I cancel plans sometimes because I don’t want to be the person constantly blowing their nose.”* (P17) *“People move away when you sneeze, it’s worse since COVID because they think you’re sick.”* (P12) *“You can’t plan anything outdoors because you never know when it’ll hit.”* (P30)
Work and Academic Impact	*“When it’s bad, I can’t think clearly I forget things and make simple mistakes.”* (P21) *“It’s hard to do your job properly when your head feels full and your eyes are watering.”* (P5) *“I work in an office, and it’s embarrassing having to keep stepping out to clear my nose, it makes you look unprofessional.”* (P28)

AR, allergic rhinitis; P, participant.

#### 3.2.5. Theme 5: Limited Awareness and Barriers in Healthcare Access Hinder Effective Management of Allergic Rhinitis

Participants described how limited awareness, inconsistent healthcare experiences and systemic barriers to healthcare access prevented effective AR management. Although widely perceived as a minor condition due to its prevalence, misinformation, inadequate clinical engagement and cost significantly hindered long-term care.

##### Awareness and Knowledge Gaps

Participants described a pervasive lack of awareness about the chronic nature of AR, both among the general public and individuals living with the condition: *“People think it’s just hay fever, but they don’t see the exhaustion or the effect on your life”* (P4). Limited health literacy often led to self-diagnosis and inconsistent management through trial and error.

##### Experience with Healthcare Professionals

Interactions with healthcare professionals varied widely, with participants expressing frustrations at the perceived superficiality of care: *“Every appointment is the same ‘take antihistamines, use a spray’ but no one talks about why it keeps happening”* (P8). Some described positive experiences with specialists, noting that allergists provided more personalised management plans, though such encounters were exceptions due to referral and access barriers.

##### Healthcare System Barriers

Systemic issues, particularly cost, access and resource availability further constrained effective management. The high cost of medications and consultations was a recurring concern: *“You end up choosing between buying sprays or paying other bills, it shouldn’t be that expensive to breathe properly”* (P6). Long waiting periods for specialist appointments, particularly in the public system, were perceived as an additional barrier to timely specialist access. 

At the centre of these accounts was a disconnect between AR’s perceived triviality and its substantial lived impact, one that shaped every level of care participants encountered. Additional illustrative quotes supporting Theme 5 are presented in Table 5.

**Table 5:** Supporting participant quotes for Theme 5: Limited awareness and barriers in healthcare access.

Sub-Theme	Participant Quotes
Awareness and Knowledge Gaps	*“I didn’t even know there were pollen forecasts or specialist clinics until years after being diagnosed.”* (P11) *“Didn’t realise headaches/fatigue were part of hay fever until recently. That was a new revelation.”* (P18) *“You end up trying every over-the-counter tablet and hoping something works.”* (P19)
Experience with Healthcare Professionals	*“Doctors treat it like an inconvenience, not something that actually affects your sleep or concentration.”* (P13) *“GPs are dismissive because allergies are common.”* (P7) *“It took a long time for doctors to call it hay fever, it was always treated like something temporary.”* (P18) *“Doctors mostly prescribed steroids for my eczema and antihistamines for hay fever. Sometimes I feel they’re just throwing meds at me without long-term solutions.”* (P27) *“There’s a lot of mismanagement and bad advice…pharmacists recommending vasoconstrictor drops, which is bad for the eyes.”* (P28) *“It wasn’t until I saw an allergy specialist that I understood what triggers me, but getting that referral took months.”* (P21)
Healthcare System Barriers	*“Immunotherapy worked for me, but I had to stop because it was too costly”* (P30) *“Currently on immunotherapy…costs around $400 a year, plus approximately $100 for antihistamines”* (P29) *“I waited nearly a year to see an allergist, by then the worst season was already over.”* (P23) *“Private access is easier; public has 6 to 12-month waits.”* (P29) *“If you can’t afford private care or live in the city, you’re stuck managing it on your own.”* (P17) *“Most of us just do what we can; buy what’s available, avoid triggers, and hope for the best.”* (P10)

GP, general practitioner; P, participant.

## 4. Discussion

This study explored the lived experiences of adults with AR in urban Victoria, Australia, including both self-diagnosed and professionally diagnosed participants, and to our knowledge represents the first qualitative inquiry into this population within this context. Five themes emerged, covering origins, environmental, management, psychosocial and healthcare system dimensions of AR. The Wilson and Cleary model of HRQoL served as the organising framework, mapping a conceptual pathway from biological and clinical variables through symptom status, functional status and general health perceptions, to overall HRQoL [34,40,41]. This framework is particularly well suited to AR, where allergen-driven nasal inflammation produces symptoms that ripple outward to impair sleep, work, social functioning and perceived wellbeing beyond clinical indicators alone [41,42,43]. Qualitative methods are well positioned to illuminate this pathway as lived experience, capturing experiential processes, contextual factors and personal meanings that standardised instruments cannot fully detect. Table 6 summarises selected qualitative and patient-experience literature and contextualises the findings of the present study within this field.

More than half of the participants were self-diagnosed, with accounts describing recurrent symptoms that had previously been attributed to colds or seasonal irritants, reflecting a broader pattern in which AR goes unrecognised as the chronic condition it is, both by those living with it and by those around them, including family, friends and the wider healthcare system. This is consistent with Australian evidence showing that AR patients frequently move into unguided self-management before ever receiving a formal diagnosis [19,29]. The high rate of self-diagnosis among participants is an important finding, reflecting a pathway in which AR’s gradual onset and accessible over-the-counter treatments may encourage self-recognition and self-management, particularly when formal diagnosis may be viewed by some patients as adding limited value beyond a diagnostic label. However, findings relating to the self-diagnosed participants are interpreted in the context of their reported experiences rather than formal diagnostic confirmation. Across participants, self-diagnosis, trial and error management, and a normalising disposition toward ongoing symptoms formed a consistent pattern. Among participants with a professional diagnosis, persistent symptoms may be compatible with ongoing allergic inflammation; however, inflammatory activity was not directly assessed in this study. The long-term consequences of chronically undertreated AR remain poorly understood; however, persistent type 2 inflammatory signalling has been linked to lower airway disease and sleep-disordered breathing [48,49], raising the possibility that ongoing unmanaged inflammation may carry broader respiratory and sleep-related consequences over time in this subgroup.

Beyond its established role as a biological risk predictor, family history functioned as an informal health literacy system, as reflected in participants’ accounts of inheriting practical routines and coping knowledge from allergic family members. Participants from allergic households inherited a practical framework for managing AR through social learning processes, whereas those without this background lacked equivalent scaffolding [50,51]. Clinicians should not assume equivalent baseline understanding at diagnosis as patients without a family history of atopic disease may need more deliberate structured support, including guidance on first steps, treatment escalation and when to return. AR should be approached as a chronic condition requiring ongoing management rather than a recurring complaint resolved by a single prescription [52,53,54].

Several participants described developing AR-consistent symptoms after relocating to urban Victoria. While causality cannot be established from qualitative accounts, this pattern raises a hypothesis that environmental exposures associated with urban Victoria may be associated with symptom onset or worsening of symptoms in some new residents [55,56,57]. This hypothesis requires further epidemiological investigation.

Participants also described symptoms occurring indoors, attributing these experiences to factors such as dust mites, pet dander and mould, which they perceived as triggering symptoms as readily as outdoor allergens and contributing to a pervasive psychological burden of having nowhere to retreat. These accounts challenge the practical assumption underpinning current avoidance guidelines that the home is a controllable environment [58,59] and are consistent with evidence of disproportionate symptom burden even in buildings meeting standard air quality thresholds [60,61]. Australia currently lacks allergen-specific indoor air quality guidelines [62,63]. Participants’ accounts raise the question of whether greater consideration of allergen-aware indoor air quality guidance may be warranted.

While sleep disruption and AR-related presenteeism are well documented [21,64,65], this study adds an identity dimension that is largely absent from existing data. Cognitive fog, repeated errors and missed practical training threatened how participants perceived themselves as competent workers and students, representing a quiet erosion of occupational self-image that productivity metrics cannot capture [66,67]. This finding strengthens the case for routine assessment beyond symptom scores in AR consultations. Furthermore, participants in this study described a form of social stigma introduced by the COVID-19 pandemic, whereby AR symptoms such as sneezing and coughing were misread by others as signs of infectious illness. Participants described being perceived as infectious when sneezing publicly, withdrawing socially to avoid explanation, and extending environmental vigilance beyond allergen monitoring into social settings. This post-COVID stigma appears to compound the pre-existing social burden of AR and warrants further investigation. These findings suggest that AR consultations may benefit from routine assessment of emotional wellbeing and social functioning, with clinicians asking directly whether visible symptoms are causing social anxiety or avoidance. Public health messaging during peak pollen seasons could also consider distinguishing allergic from infectious respiratory symptoms.

**Figure 2:**
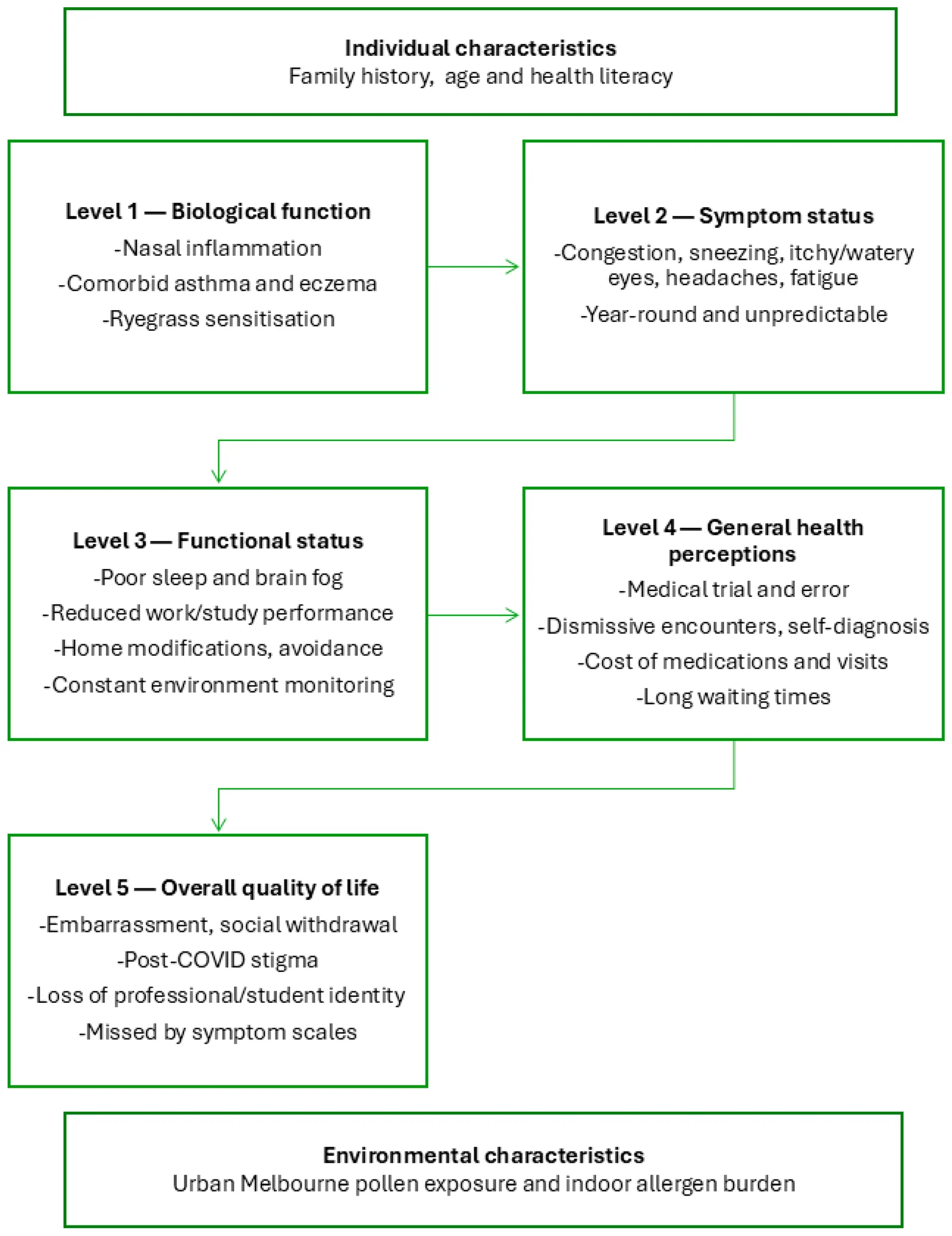
The Wilson and Cleary model of health-related quality of life applied to the lived experience of allergic rhinitis in urban Australia. Biological-function elements are conceptual and do not represent measurements obtained for all study participants.

Mapped onto the Wilson and Cleary model (Figure 2), participants’ reported AR symptoms and experiences cascaded through sleep disruption, cognitive fog and environmental vigilance into eroded occupational identity, emotional distress and post-COVID social stigma, while episodic clinical encounters reinforced cycles of self-management and cumulative burden [34,41]. Standardised instruments, such as the RQLQ, capture symptoms and function at a single timepoint but may not fully capture these cumulative, identity-threatening and socially embedded experiences [68,69], supporting qualitative inquiry as a valuable complement to Patient-Reported Outcome Measures in both AR research and clinical assessment [70,71].

### Strengths and Limitations

To our knowledge, this is the first qualitative study focused specifically on the lived experience of adults with AR in urban Victoria, extending prior Australian qualitative research on AR management and treatment-seeking [19,29,44]. This study’s urban Victorian focus may not reflect rural or regional experiences, and the sample was predominantly female (68.4%, *n* = 26), which may limit transferability to male populations. The sample also had a relatively young mean age (31.4 years), which may limit transferability to older populations. Demographic and clinical characterisation beyond age, gender and diagnostic status was not systematically collected and is not reported quantitatively. Although the inclusion of self-diagnosed participants was important for capturing the experiences of individuals who may not seek or receive formal diagnosis, 57.9% (*n* = 22) of the participants did not have a professional diagnosis. The absence of structured diagnostic verification means that alternative causes of rhinitis-like symptoms could not be systematically excluded, introducing some diagnostic uncertainty. Furthermore, although multiple recruitment channels were used, including flyers, social media, word-of-mouth and snowball sampling, the use of social media and snowball recruitment may have introduced some selection or homogeneity bias. 

Interviews were conducted between June and October, spanning winter and spring, which may have shaped which symptoms and experiences were most salient to participants. As the interview guide was informed by the Wilson and Cleary model, the resulting themes may have been shaped by this framework’s domains, although coding and theme development were conducted inductively. Finally, as a qualitative study, this research cannot establish causal environmental or biological relationships; related observations should be understood as hypotheses generated by participants’ accounts rather than established findings.

Future research comparing urban and rural Victorian experiences, with more systematic demographic and clinical characterisation, would help form a more complete picture.

## 5. Conclusions

Participants described AR as extending beyond physical symptoms, with emotional, social and economic impacts, and as a chronic, cumulative condition in which environmental exposure, sleep disruption, emotional strain and inconsistent healthcare support interact to erode QoL in ways that single-timepoint measures may not fully capture. This study identifies family history as an informal health literacy system, blurred indoor-outdoor boundaries as a barrier to avoidance, and links between sleep disruption and identity-related impacts, alongside an emerging, under-recognised burden of post-COVID social stigma. These findings reinforce the need to integrate lived experience alongside clinical indicators in AR research and care planning, which may benefit clinical practice.

## Figures and Tables

**Table 6:** Qualitative and patient-experience literature on allergic rhinitis and comparison with the present study.

Areas Examined and Key Findings	Study 1 (Cvetkovski et al., 2018) [19]	Study 2 (Cvetkovski et al., 2019) [44]	Study 3 (Jacob et al., 2025) [29]	Study 4 (Cottet et al., 2025) [45]	Study 5 (Noreña-Peña et al., 2025) [46]	Study 6 (Belins et al., 2022) [47]	Our Study
Areas Examined							
Diagnosis/symptom experience	✓	✓	✓	✓	✗	✓	✓
Environmental/seasonal triggers	✗	✗	✗	✗	✗	✓	✓
Coping and management strategies	✓	✓	✓	✓	✓	✓	✓
Financial burden	✓	✗	✓	✓	✗	✗	✓
Social burden/stigma	✓	✗	✓	✓	✗	✓	✓
Emotional burden	✓	✓	✓	✓	✓	✓	✓
Sleep disruption	✗	✗	✗	✗	✗	✓	✓
Healthcare system barriers/access	✓	✓	✓	✓	✓	✗	✓
Physician–patient relationship/communication	✗	✓	✗	✓	✓	✗	✓
Daily activity limitations	✓	✗	✓	✓	✓	✓	✓
Key Findings							
Family history as informal health literacy	✗	✗	✗	✗	✗	✗	✓
Identity erosion	✗	✗	✗	✗	✗	✗	✓
Post-COVID social stigma	✗	✗	✓	✗	✗	✗	✓

✓ = area examined or reported in the cited article; ✗ = not identified or reported in the cited article. This table represents a narrative comparison and is not intended as a systematic assessment of study quality or research gaps.

## Data Availability

The data associated with this manuscript are available from the corresponding author upon reasonable request.

## Supplementary Materials

The following supporting information can be downloaded at: https://www.mdpi.com/article/10.3390/jcm15187284/s1, Supplementary Material S1: Semi-Structured Interview Guide; Supplementary Material S2: COREQ (COnsolidated criteria for REporting Qualitative research) Checklist.

## Abbreviations

The following abbreviations are used in this manuscript:

AR	Allergic rhinitis
ARIA	Allergic Rhinitis and its Impact on Asthma
COREQ	Consolidated Criteria for Reporting Qualitative Research
GP	General practitioner
HREC	Human Research Ethics Committee
HRQoL	Health-related quality of life
QoL	Quality of life
RQLQ	Rhinoconjunctivitis Quality of Life Questionnaire
RTA	Reflexive thematic analysis
