# Supplementary material for: The Impact of Allergic Rhinitis on Australian Adults in an Urban Setting: A Qualitative Exploration
Source: J Clin Med. 2026 Sep 19;15(18):7284. doi: 10.3390/jcm15187284 (PMC13607325; doi:10.3390/jcm15187284)
Supplement: Supplementary file 1 [file jcm-15-07284-s001.zip › Supplementary Material S1_Interview Guide.pdf]

Semi-Structured Interview Guide

Exploring the Allergy Burden: A Study on Australian Adults

The questions outlined in this interview guide serve as a framework to ensure alignment with the study's aims and objectives. While the general structure will be adhered to, the interviewer may adapt, skip, or expand upon specific questions based on the participant's responses. This approach allows the conversation to remain natural and ensures a smooth flow, enabling participants to share their experiences and perspectives more comfortably and comprehensively.

Introduction and Consent

Welcome and Rapport Building:

- Hi [Name], how are you today? (Not recorded during initial greeting)
- Thank you for agreeing to participate in this study. Your insights are invaluable to us.
- Before we start, can you please complete the questionnaire by clicking on the link in the chat?
- Is it okay if I start recording now? (Wait for affirmation)
- *Begin recording*
- Thank you. I'll now turn on the recording.

Consent and Study Overview:

This study explores the impact of allergic rhinitis and ocular allergies on various aspects of life, including physical, emotional, and social dimensions.

The interview will last about 60 minutes, and all responses will remain confidential. No names or identifying information will be included in transcripts. Does this sound okay? (Wait for confirmation)

You can skip any question or withdraw at any time during the interview. After transcription, withdrawal will no longer be possible due to anonymisation.

Do you have any questions before we begin?

Section 1: Demographics and Background

Purpose: Understand participant context, including geography and health factors.

1. Can you tell me a little about yourself? (Prompts: age, occupation, cultural background, hobbies)
2. Where do you currently live? Is it an urban or rural area?
   - Have you always lived in this type of setting?
3. How would you describe your overall health? (Prompt: Any chronic conditions or family history of allergies? Asthma? eczema?)

Section 2: Symptom History and Diagnosis

Purpose: Capture allergy symptom patterns, triggers, and diagnosis experiences.

4. What type of allergy do you have? How was it diagnosed?
5. When did you first notice your allergies?
6. What are your main triggers? (e.g., pollen, dust, food)
   - For pollen: "Do you check pollen count websites? How does this influence your activities?"
7. Can you describe your symptoms and their severity? (Prompt- eye symptoms)
8. Do they change with seasons or environments?
9. Do your symptoms worsen during specific seasons, such as spring?
   - If you think about a typical week during your allergy season, on how many days do you usually experience symptoms like sneezing, congestion, or itchy eyes

- Roughly how many weeks do you feel your symptoms are present or affecting you?

10. Have environmental factors like pollution or bushfires affected your symptoms?

Section 3: Environmental and Geographic Factors

Purpose: Examine geographic and environmental influences.

11. How does seasonal weather affect your symptoms?
12. Do you notice differences in symptoms severity based on where you live or travel (urban vs rural)?
    - Which places have you travelled to?

Section 4: Emotional and Social Impact

Purpose: Explore emotional and social consequences of allergies.

13. How do your allergies affect you emotionally?
14. Have they impacted your social life or family activities?
    - Can you give me an example of a time your symptoms affected plans with friends or family?
    - Have you ever had to cancel or avoid any gatherings because of your symptoms?
15. How do your family or close friends react or respond when your symptoms flare up?
    - Do you ever feel you need to explain your symptoms to others in social settings?
    - Do others mistake your allergies with the flu?

Section 5: Work, Productivity, and Lifestyle

Purpose: Investigate practical impacts on daily functioning.

16. Have your allergies affected your day-to-day activities? Eg: career choice, school productivity or productivity at work?
17. Do you need to take time off or adjust your tasks due to symptoms?
18. How does it impact your sleep?
    - Do you sleep more or less when symptoms are present?
19. What's the most challenging or upsetting experience you've had because of your hay fever?

Section 6: Coping Strategies and Treatment *(Aim 4)* Purpose:
Assess management strategies and barriers.

20. What treatments or strategies have you tried, and how effective are they?
21. What made you pick up these medication/who recommended these treatments (prompt- GP, specialists, friends, online channels, pharmacists etc.)
22. How do you access these medications, and which works best (prompt-prescribed or over the counter)
23. Have you experienced challenges in accessing or affording treatment?
24. How much do you typically spend on medication during peak symptom season?
25. How do you manage persistent symptoms, and how does this affect you?

Section 7: Healthcare Experience *(Aim 3)*

Purpose: Understand healthcare access and support.

26. Do you feel supported by healthcare professionals in managing your condition?
27. How easy is it to access healthcare for your allergies?

Section 8: Future Aspirations and Suggestions ***(Aim 5)***
Purpose: Gather participant-driven insights for better management.

28. If you could share one message with healthcare providers or policymakers, what would it be?

Closing the Interview

29. We've covered a lot today. Is there anything else you'd like to share about your allergy experience? (Open ended and do not interrupt participant).

Thank you so much for your time and insights! If you have any additional thoughts later, or know someone else who might want to participate, please let us know."
